# Manually compressed soil blocks stabilised by fly ash based geopolymer: a promising approach for sustainable buildings

**DOI:** 10.1038/s41598-023-50103-6

**Published:** 2023-12-21

**Authors:** Quoc-Bao Bui, Tan-Phat Nguyen, Dirk Schwede

**Affiliations:** 1 Sustainable Developments in Civil Engineering Research Group, Faculty of Civil Engineering, Ton Duc Thang University, Ho Chi Minh City, Vietnam; 2 Energy and Building Services, Department of Architecture and Civil Engineering, Technische Hochschule Lübeck, Lübeck, Germany

**Keywords:** Civil engineering, Environmental impact

## Abstract

The construction industry is one of the sectors which have significant impacts on the environment. The research on sustainable materials is a demand of society. This paper presents an investigation on the use of fly ash (FA) geopolymer binder for the production of unburnt bricks. First, an optimisation process for the ratio of alkaline activator solution (AAS) and FA was performed. The blocks were obtained by compressing the materials in a mould by hand, similar to the traditional technique of the adobes. Different ratios of AAS in the blocks were investigated: 6%, 8%, 12% and 20% by mass, respectively. Two curing temperatures were tested: ambient temperature and at 60 °C. Then, different properties of the blocks were determined: flexural tensile strength, compressive strengths (in the quasi-dry state and in the saturated state), water absorption. The techniques of Fourier Transform Infrared (FTIR) and Scanning Electron Microscope (SEM) were also used for the analyses of the results obtained. The results showed that the blocks with 20% AAS had highest compressive strengths with an average of 24 MPa at 28 days, while the recommended AAS amount for both technical and economical points of view was 8%, with a mean compressive strength of 13 MPa at 28 days. The ratio between the saturated compressive strength on the quasi-dry compressive strength was higher than 0.5, which satisfied the current exigencies from the standards. These exploratory results are important for practice applications of this type of blocks.

## Introduction

Building with earth is an ancient technique which has been used by human since millenniums. Different techniques of earth construction exist: rammed earth, cob, adobes, compressed earth blocks (CEB)^1^. Earth materials have low embodied energy^2,3^ and positive hygro-thermal behaviour^4–6^. However, with the apparition of industrial materials (such as concrete), the use of earth materials has significantly decreased. Nevertheless, cement concrete, with the use of aggregates and Ordinary Portland Cement (OPC), is criticized as contributing to the natural resource depletion, the high energy consumption and the CO_2_ emission^7–9^. The over-exploitation of river sand provokes several environmental problems, especially the erosions and the sliding of the riversides. The river sand becomes progressively a rare resource for the construction projects when the number of new constructions is increasing. Therefore, alternative solutions to replace river sand and OPC are urgently demanded.

While the natural gravel in cement concrete can be partially or totally replaced by coarse recycled aggregates, the natural river sand can only be partially replaced by fine recycled aggregates^10^. The soil was proposed to totally replace the gravel and sand^11–13^. A “renaissance” of earth construction is observed during the last decade in the context of a circular economy^14^. However, the modern earth materials must follow the modern standards which have been developed for industrial materials^14^. One of the limitations of earth materials is the sensibility to water^15^. To enhance the durability of earth materials, hydraulic binders (cement or lime) are usually proposed^1,15–20^. Geopolymer is also recently proposed as a stabiliser for earth materials^21–32^. Indeed, geopolymer is considered as a promising alternative binders for sustainable constructions^33^, and also for future constructions on Moon^34^. Several studies showed that geopolymer had less environmental impacts than cement^35,36^. Although geopolymer has been applied in numerous studies for the manufacturing of gepolymer concretes^37–39^, the composition of geopolymers has not yet been standardised like cement concretes. It has been observed that the alumina and silica in the soil are in crystal phase^40^, therefore, additional alumino-silicate precursors must be added (e.g. fly ash, FA). The curing at elevated temperatures was also proposed (from 60 to 450 °C) to enhance the geopolymerisation^39,41,42^.

The existing studies in the literature have investigated the geopolymer stabilisation for rammed earth^21,22^, compressed earth blocks (CEB)^23–27^ or compacted earth specimens manufactured in laboratory^28–32^. To our knowledge, no study has been carried out on the application of FA based geopolymer in the stabilisation of adobes. In fact, adobes are the blocks which are obtained by compressing the earth by hand in a wooden mould. The soils used for adobes are usually clayey soils (which are usually called “earth”).

In the present study, the blocks were manufactured by a manual compression (by hand) in a wooden mould. This manufacturing process can be easily applied for the zones where the specific mechanical machines are not available such as the rural regions or on other planets^34,43^.

The soil used in the present study was a sandy soil, which was different than the traditional adobes. The proposed strategy was to use the local soils for the manufacturing of unburnt bricks. It is important to note the willingness of Vietnamese government in the replacement of conventional clay burnt bricks by unburnt bricks, to reduce the CO_2_ emission. It is also worth mentioning that the lack of river sand is currently a crisis for the constructions in Vietnam. So, the study proposed a potential measure for these problems.

The FA geopolymer was used as a binder for the blocks in the present study. The FA has been chosen because it is a by-product generated from the coal power plants which are numerous in Vietnam. FA can be used for some applications such as concrete manufacturing or the cement industry, however, the amount of FA generated in Vietnam is higher than these consumptions. The disposal of the FA at coal power plants causes environmental problems. Therefore, the valorisation of FA is an important topic in Vietnam. The use of FA for the creation of geopolymer is an encouraging approach.

Different mechanical properties of the geopolymer stabilised adobes have been investigated: the flexural tensile strength, the compressive strength, the ratio of saturated compressive strength on the quasi-dry compressive strength. This exploratory study can open for further studies in this topic.

## Experimental investigation

### Materials used

#### Soil

The soil was extracted from a construction site located in Ho Chi Minh City, Vietnam. The size distribution testing was performed by the granulometry test (for the particles ≥ 80 μm) and the sedimentometry test (for the particles < 80 μm). The results showed that the soil had 3% clay (< 0.002 mm), 20% silt (0.002–0.05 mm), 65% sand (0.05–2 mm) and 12% gravels (> 2 mm). The initial idea of this study was to apply the geopolymer and the adobe technique for local soils (any soil). So, this soil has been taken from a construction site on the campus where several underground spaces (for the parkings) have been built. The soil extracted (for the underground stories) represented the South zone of Ho Chi Minh City, South of Vietnam. Therefore, although the soil was not adapted for traditional unstabilised adobes (where the clay amount is higher), we would like to test this soil for adobes stabilised by geopolymer.

The Atterberg limits have also been determined for the soil used following the Casagrande cup method^44^. The Liquidity limit w_L_ was of 19.8%, while the Plasticity limit w_P_ was very close to w_L_. This meant that the soil had a very low plasticity. By using the classification following the standard^44^, the soil was in class B5, corresponding to a «sandy soil».

Due to the low clay content of this soil, the approach using unstabilised soil materials (without additional binder) was not reasonable. Therefore, in the present study, the soil has been stabilised by geopolymer.

#### Fly ash

The FA used came from the DH3 coal power plant, in the South of Vietnam. The chemical composition of the FA used was determined by the EDS (energy dispersive spectroscopy) technique^45^; the results are presented in Table 3. The mineralogical composition of FA was also determined by using the XRD (X-Ray diffraction) technique^46^, the result is summarized in Table 1. From this table, the total amount of the major components SiO_2_, Al_2_O_3_ and Fe_2_O_3_ in FA is 83.65%. From Table 2, the mineralogical composition of FA is mostly amorphous phase which takes 87% of FA composition. From these results, the FA used is classified as class F fly ash in accordance with ASTM C618^45^. The amount of Al_2_O_3_ and SiO_2_ were high enough for the alkali-activation. So, the FA used was suitable for the creation of geopolymer.

**Table 1:** Chemical composition of FA used.

Components	% in mass
Sulfur trioxide (SO_3_)	1.0
Aluminum oxide (Al_2_O_3_)	26.1
Ferric oxide (Fe_2_O_3_)	11.3
Sodium oxide (Na_2_O)	1.35
Silicon dioxide (SiO_2_)	51.1
Potassium oxide (K_2_O)	1.29
Calcium oxide (CaO)	4.7
Magnesium oxide (MgO)	1.7
Moisture	0.1
Loss on ignition	0.7

**Table 2:** Mineralogical composition of FA.

Crystal phase (Quartz, Mullite, Maghemite)	13%
Amorphous phase	87%

**Figure 1:**
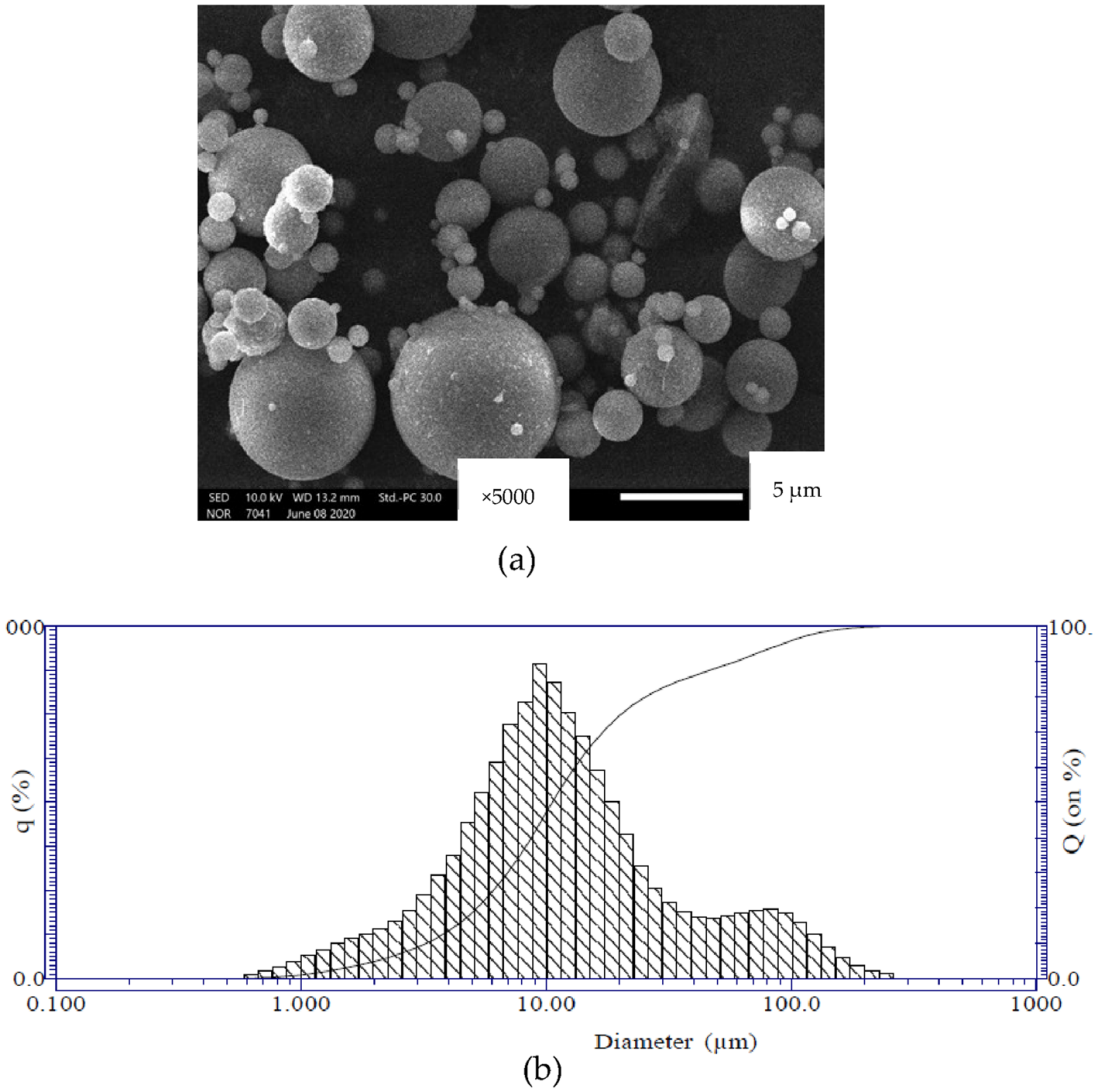
(**a**) Microstructure of the FA used, obtained by SEM; (**b**) particle size distribution of FA.

The analyses of the particle size distribution by scanning electron microscope (SEM, JSM-IT200, Jeol) showed that the FA used had spherical forms (Fig. 1a) with dimensions varying from 0.6 to 250 µm, the mean dimension was about 10 µm (Fig. 1b). The specific density of FA was also measured, giving a value of 2.44.

#### Alkaline activator solution (AAS)

A combination of sodium hydroxide solution (NaOH) and sodium silicate solution (Na_2_SiO_3_) was chosen as the alkaline activator solution (AAS) to activate FA and obtain the geopolymer. These substances have been currently used in previous studies to produce geopolymer^31,32,38,47^.

In the present study, the sodium hydroxide was in pellet form with 96% purity, a specific gravity of 2.13 g/cm^3^ at 20 °C. The Na_2_SiO_3_ solution used was a commercial product with 11.8% Na_2_O, 29.5% SiO_2_ and 58.7% water, a specific gravity of 1.44 g/cm^3^ at 20 °C. It was observed that the effect of Na_2_O/Si_2_O molar ratio in Na_2_SiO_3_ on the compressive strength of geopolymer concrete was negligible^48^. The NaOH solution was prepared by dissolving the pellets in water. The mass of NaOH solids in a solution varied depending on the concentration of the solution expressed in terms of molar, M. It was shown in previous studies that when the molar concentration of NaOH increased, the compressive strength of geopolymer-based material increased^24,26,47–51^, with the best values were in the range from 10 to 14 M^26,49^. That was why the previous studies usually used 6, 8 or 10 M of NaOH^51^, although the studies on lower molarity such as 4 M existed also^50^. In the present study, a preliminary investigation has also been caried out on the mortar specimens (sand + geopolymer) with the molar concentration of 6, 8 and 10 M, respectively. The results confirmed that the mortar specimens with 10 M had the highest compressive strength. Therefore, in the present study, the molar concentration of NaOH solution was chosen at 10 M. This concentration was an acceptable compromise between the technical and economic aspects. In fact, a higher molar concentration increases the cost, the environmental impact and also the practice application of the solution. The application of a high molar concentration solution needs several precautions due to the safety reason.

First, the NaOH pellets were dissolved in water following the molar concentration required. Then, the Na_2_SiO_3_ solution was added. The alkaline activator solution (AAS) was prepared one day prior to the mixing with other materials because the preparation of AAS was exothermic. The important parameter on the compressive strength of geopolymer was the ratio between Na_2_SiO_3_ and NaOH^39,47–49^. For geopolymer mortar or concrete, it was noted in numerous studies that the ratios of Na_2_SiO_3_/NaOH from 1.5 to 3 provided the highest compressive strength^47–49^ and the usual ratios were from 2 to 2.5. In the present study, the Na_2_SiO_3_/NaOH ratio of 2.5 was used. For geopolymer concrete, AAS/FA ratio was usually taken in the range from 0.4 to 0.5 (by dry mass); the results were not significantly different in this range^39,47^. In the present study, the ratio of AAS/FA was taken at 0.5.

### Manufacturing of the blocks

#### Composition

The NaOH solution used in the study had a concentration of 10 M, corresponding to each litre of solution containing 400 g of anhydrous NaOH solid. The solution was cooled before mixing with sodium silicate (Na_2_SiO_3_) to form AAS. The ratio of FA/AAS was investigated to obtain the maximum compressive strength of geopolymer^49^. Indeed, following the existing studies in the literature, the Na_2_SiO_3_/NaOH ratio recommended for FA geopolymer was of 2.5^47,49,51^. Then, a preliminary study had been caried out on the geopolymer paste samples to determine the optimum compositions of geopolymer used in this study^39^. The results showed that the best AAS/FA ratio was 0.45–0.5 which provided the highest compressive strengths. That was why in the present study, the ratio FA/AAS = 2 was chosen.

First, the referent composition chosen was 6% AAS, because the current amounts of cement stabilisation have been 6 or 8% (in mass). This composition of 6% AAS correspond to 4.3% Na_2_SiO_3_ + 1.7% NaOH (to have the ratio Na_2_SiO_3_/NaOH = 2.5) and 12% FA (to have the ratio FA/AAS = 2), the rest was the soil. Then, other compositions were chosen: 8%, 12% and 20% AAS (in mass), to evaluate the influences of AAS on the blocks manufactured. The amount of 20% AAS was high compared to the current stabilisation ratios of soil-based materials, however, for conventional cement blocks, the cement amount of 20% is current in many cases. Therefore, in this exploratory study, a high amount of AAS was also tested.

**Figure 2:**
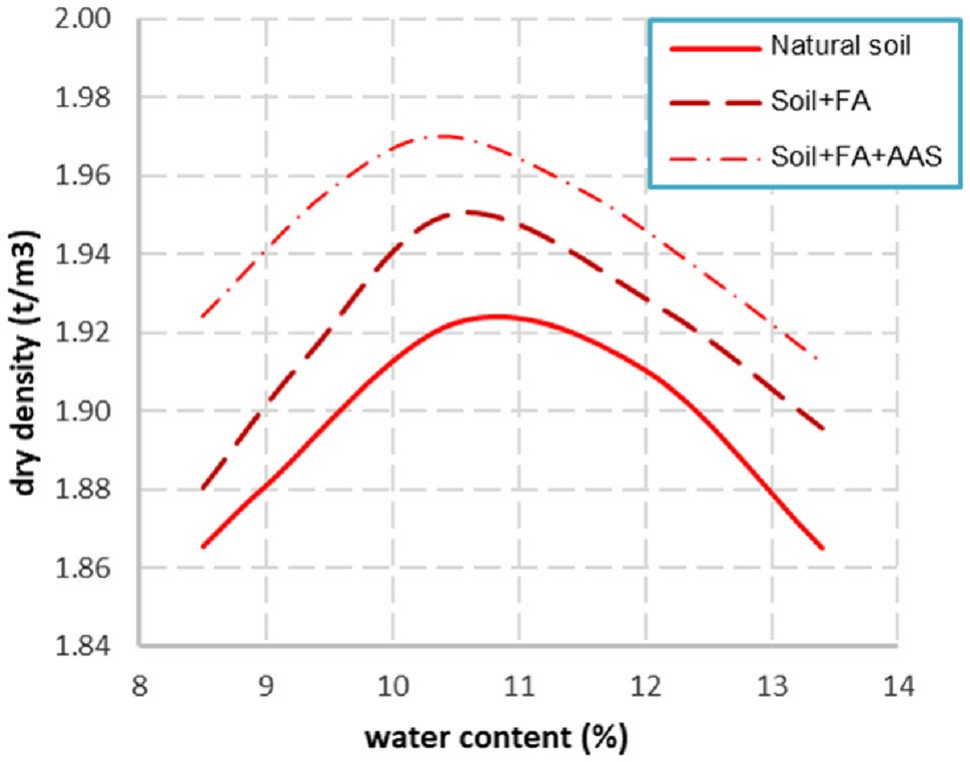
Determination of the optimum manufacturing water content for different compositions.

The manufacturing water content is an important parameter which determines the dry density and the compressive strength of soil-based materials. For rammed earth, the Modified Proctor test was proposed to determine the water manufacturing^52^. In the present study, although the manual compression energy was not the same as the case of rammed earth, the Modified Proctor tests were also carried out. The results of the dry density of the blocks obtained will be discussed later. The results are presented in Fig. 2, for different compositions: soil, soil mixed with FA, and soil mixed with FA and AAS. The water contents presented in the figure were the “total water” in the samples which were determined after the Modified Proctor tests (including water in the AAS, not only the water added). The results from Fig. 2 show that the optimum water content of the soil is 10.8%. The mixture of soil and FA has an optimum water content of 10.5% which is slightly lower than that of soil. For concrete material, it is well-known that FA can increase the workability. The higher dry density of “Soil + FA” may also due to the small size of FA particles which can fill the micropores of the material.

For the mixture of “Soil + FA + AAS”, the optimum water content is 10.3%. The lower optimum water content compared “Soil + FA” suggests that the AAS reduces the frictions between the particles. The slightly higher dry density of “Soil + FA + AAS” may be explained that the particles of Na_2_SiO_3_ and NaOH have filled the micropores of the material.

In the present study, the total manufacturing water content was chosen at 10.5%. For the compositions of “6% AAS” and “8% AAS”, the amount of additional water was calculated and the water was mixed with the soil. For the compositions of “12% AAS” and “20% AAS”, no additional water was necessary. It worth noting that the mixture of “20% AAS” had a plastic form, more likely the traditional adobes than the soil for rammed earth. The final compositions of the mixtures are presented in Table 3. The soil quantity in the Table has already included the water.

**Table 3:** Components of the different compositions investigated, in %.

Components	20% AAS	12% AAS	8% AAS	6% AAS
Na_2_SiO_3_	14.3	8.6	5.7	4.3
NaOH	5.7	3.4	2.3	1.7
Fly ash	40.0	24.0	16.0	12.0
Soil	40.0	64.0	76.0	82.0

**Figure 3:**
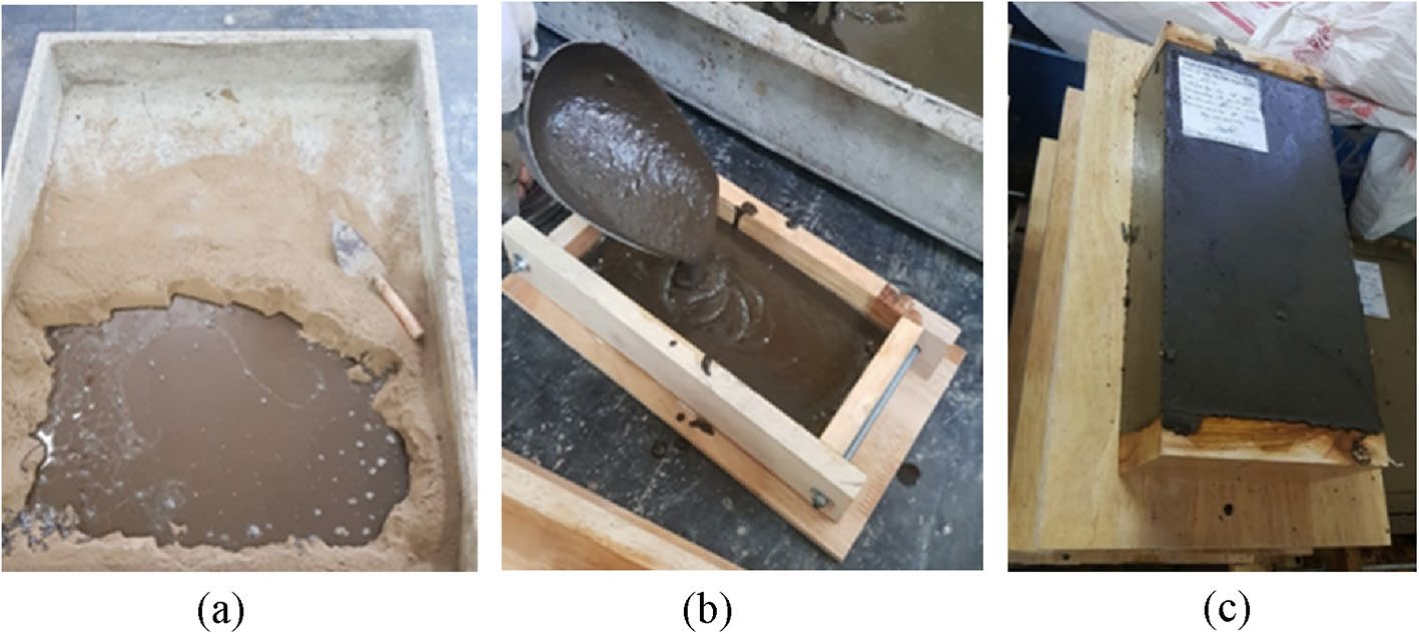
(**a**) Soil and FA were first mixed in the dry state, then AAS was added to the mixture; (**b**) placing the mixture in the mould; (**c**) block obtained after the unmoulding.

The geopolymer blocks were manufactured in the laboratory. First, the soil was humidified (when needed). Then FA was added and mixed within about 5 min (until the homogenisation of the mixture). AAS was prepared by adding NaOH solution to Na_2_SiO_3_ solution. Next, AAS was added to the mixture of soil and FA (Fig. 3a). The mixture was mixed and then put into a wooden mould with dimensions of 60 mm × 120 mm × 240 mm (Fig. 3b). Indeed, the current dimensions of the solid bricks in Vietnam are 55–65 mm thickness × 95–150 mm width × 205–250 mm length, and the ratio between these dimensions is usually 1:2:4. In the present study, the ratio 1:2:4 was also used, so the dimensions of 60 mm × 120 mm × 240 mm were chosen for the blocks. A wooden plate was put on the upper surface of the block and then the block was carefully compressed by hands. Then, the block was unmoulded immediately after the compression (Fig. 3c).

**Figure 4:**
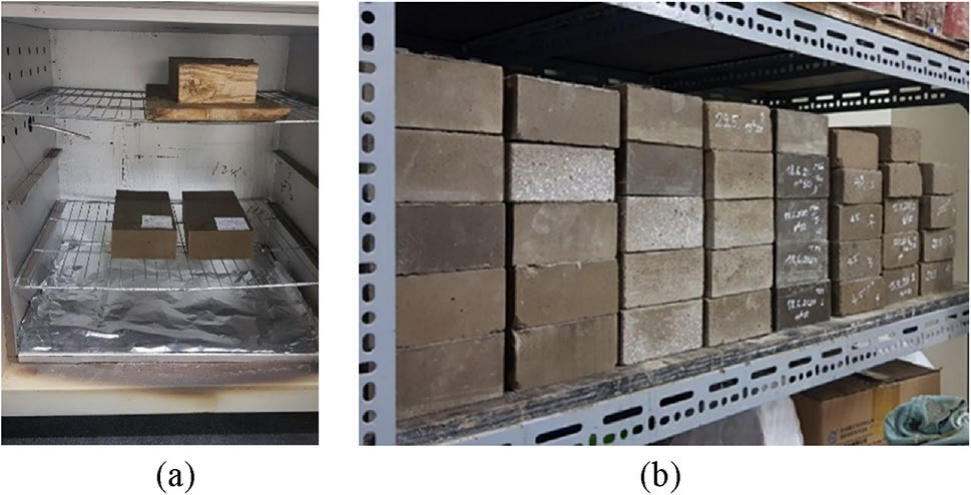
The blocks after the manufacturing, cured in the oven (**a**) and stored at room temperature (**b**).

Finally, to investigate the influences of the curing temperature on the mechanical properties of the blocks, two different curing temperatures have been tested: at ambient temperature and at 60 °C during 24 h. The temperature of 60 °C was chosen to investigate effects of the curing temperature on the material obtained. It was observed in previous studies that this curing temperature could enhance the mechanical characteristics of geopolymer^39^. Higher curing temperatures were not chosen because the curing at elevated temperatures increases the energy consumption and therefore increases the carbon footprint of the material. For specimens cured at 60 °C during 24 h, first the specimens were also kept at ambient temperature for 12 h, then cured at 60 °C in the oven for 24 h; next, these specimens were replaced at the ambient conditions of the laboratory (about 28 °C and 60%RH) (Fig. 4).

Miranda et al.^26^ compared the geopolymer stabilised CEBs with soil sieved at 4.75 mm and with soil sieved at 6.3 mm, respectively; the results showed that specimens with finer soil fraction had better compressive strengths. In the present study, the soil was also sieved at 4.75 mm to take the fine fraction. In fact, the soil did not contain significantly big grains, the sieving at 4.75 mm was to remove some coarse grains, in order to have fine elements as discussed in the previous study (Miranda et al.^26^). Moreover, the use of fine grains enables to consider the blocks obtained (60 mm × 120 mm × 240 mm) homogeneous at the macroscale.

**Figure 5:**
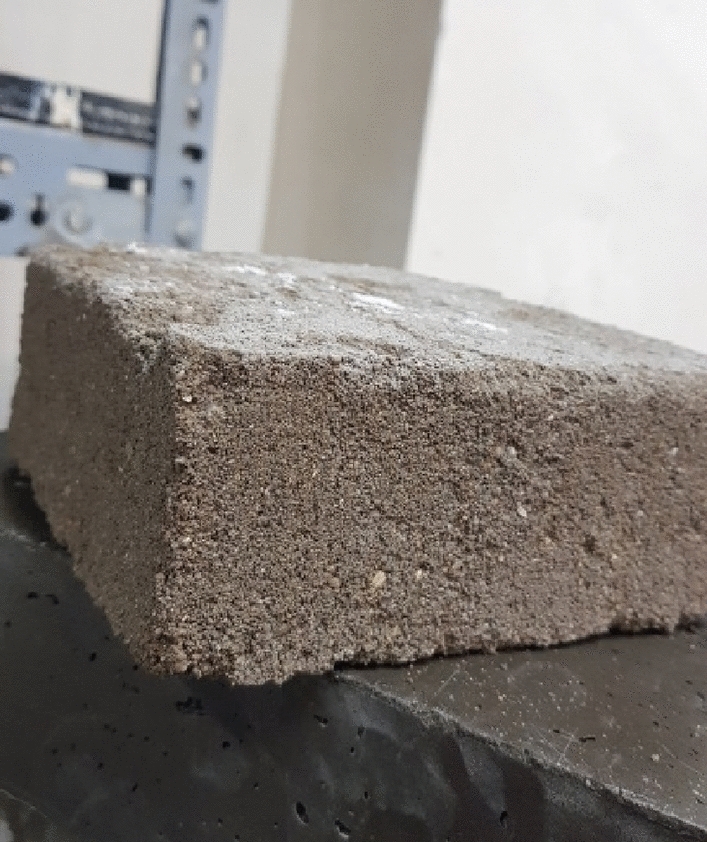
Block stabilised at 3% AAS.

The stabilisation with lower AAS (at 3% AAS) was also investigated. However, due to a low clay content of the soil used, with a low AAS, the blocks obtained did not have enough binder and did not have satisfying quality for the mechanical tests (Fig. 5). Therefore, the blocks with low AAS contents are not presented in this study.

## Material characterisation

### Three point bending tests and uniaxial compression tests

**Figure 6:**
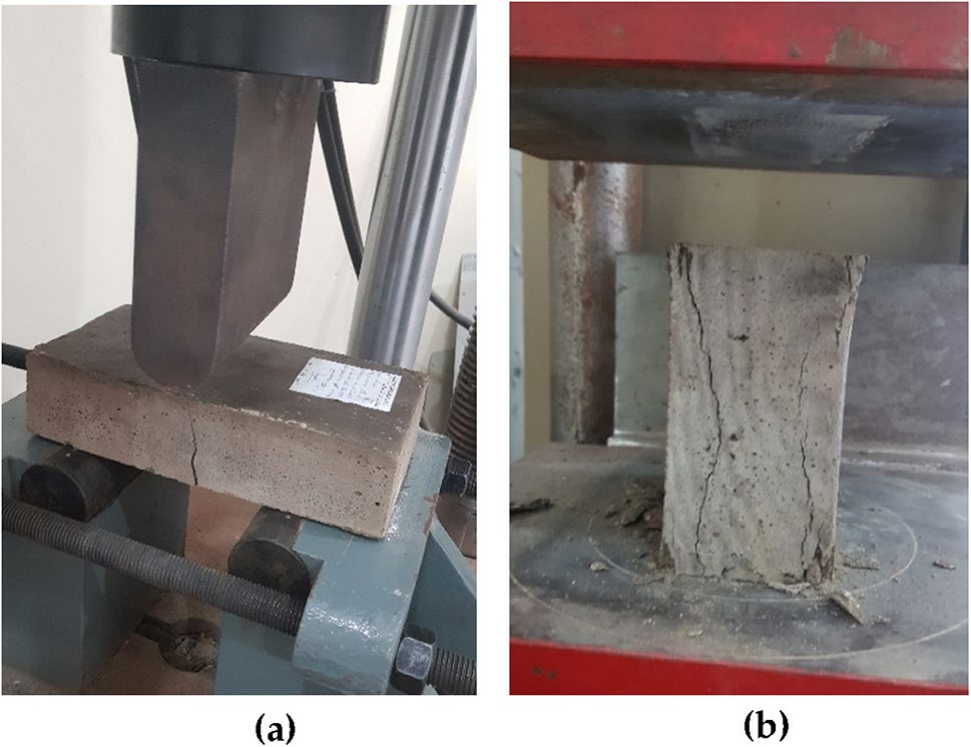
(**a**) Three-point bending test; (**b**) uniaxial compression test.

First, the blocks were tested under 3-point bending tests to determine the flexural tensile strength (Fig. 6a). Then the half-blocks were shaped and tested under uniaxial compression tests (Fig. 6b). For each experimental result, four blocks were tested under 3-point bending tests, to obtain 8 half-blocks. Then, among these half-blocks, 3 half-blocks were tested under uniaxial tests in the quasi-dry state; 3 other half-blocks were submersed in the water for 24 h and then tested in the saturated state. Two other half-blocks were used for the water absorption tests. It was observed that the water absorption values did not significantly vary between the blocks of the same type, so two tests were adopted. The blocks were tested at 7, 14 and 28 days after the manufacturing, respectively. The moisture content was determined after the uniaxial compression tests, from which the dry density was determined.

### Water absorption

The water absorption tests were performed for the samples at 28 days. The samples were immersed in water for 24 h, then the mass of the water absorbed was determined.

### SEM and FTIR tests

The microstructure and the chemical reactions of the blocks were investigated by using the Scanning Electron Microscope (SEM) and Fourier-Transform Infrared Spectroscopy (FTIR) tests. For SEM tests, the specimens were broken from the centre into small fragments. Prior to SEM tests, the specimens were coated with platinum which is a metal coating, to create a conductor layer on the specimens, to enhance the quality of the images. Then, the SEM analyses were performed.

FTIR was used to identify chemical bonds and structural changes in materials by producing an infrared absorption spectrum^53^. The spectra produce a profile for the specimen tested a distinctive molecular fingerprint that can be used to identify components in the specimen. Indeed, the vibrating bonds in functional groups absorb energy at a frequency that corresponds to the vibrational frequency of the bond; these frequencies are expressed as wavenumbers which are calculated by the ratio between the frequency on the speed of light. Within a narrow range, each wavenumber corresponds to a type of bond.

## Results and discussion

### Compressive strength

**Figure 7:**
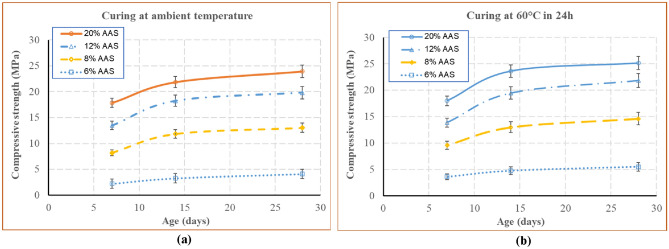
Compressive strength as a function of time for the blocks cured at ambient temperature (**a**); 60 °C during 24 h (**b**).

The synthesis of the results from the uniaxial compression tests is presented in Fig. 7. This figure shows the evolution of the mean compressive strength as a function of time. For both curing conditions (under ambient condition and at 60 °C for 24 h), the evolution of compressive strength was rapid in the first days and then slow down from 14 days. The blocks cured under 60 °C for 24 h had higher compressive strength (about 5%) than that of the blocks cured under ambient temperature.

The current compressive strengths of soil-based materials were in the range from 0.5 to 8 MPa, depending on the type and the amount of stabiliser^1^. In the previous studies^26,31^ which used FA based geopolymer for the stabilisation of clayey soils, compacted following rammed earth or CEB process, the compressive strengths obtained were less than 10 MPa. The higher compressive strength of geopolymer stabilised soil (more than 10 MPa for the curing at the ambient temperature) was observed in a previous study^27^. However, in this previous study, the geopolymer was developed by using metakaolin and rice husk ash. Moreover, the samples were the standard mortar samples, which were not the blocks at the real scale. The present study has worked on the manual compressed blocks using a soil stabilised by FA based geopolymer. The high compressive strengths obtained in the previous study^27^ and the present study suggest that for soil blocks stabilised by geopolymer, the moulding of the soil at a plastic state (like adobe) is more suitable than that of the dry state (like rammed earth or CEB). It is suggested that at a plastic state, the mixture could better fill the formwork and the micro-voids of the material. It is also suggested that the sandy soil may be more suitable for geopolymer stabilisation than clayey soil, because there can be some competition process between clay particles and the geopolymer structure at the microscale. These results should be verified by further studies on different other soils.

**Figure 8:**
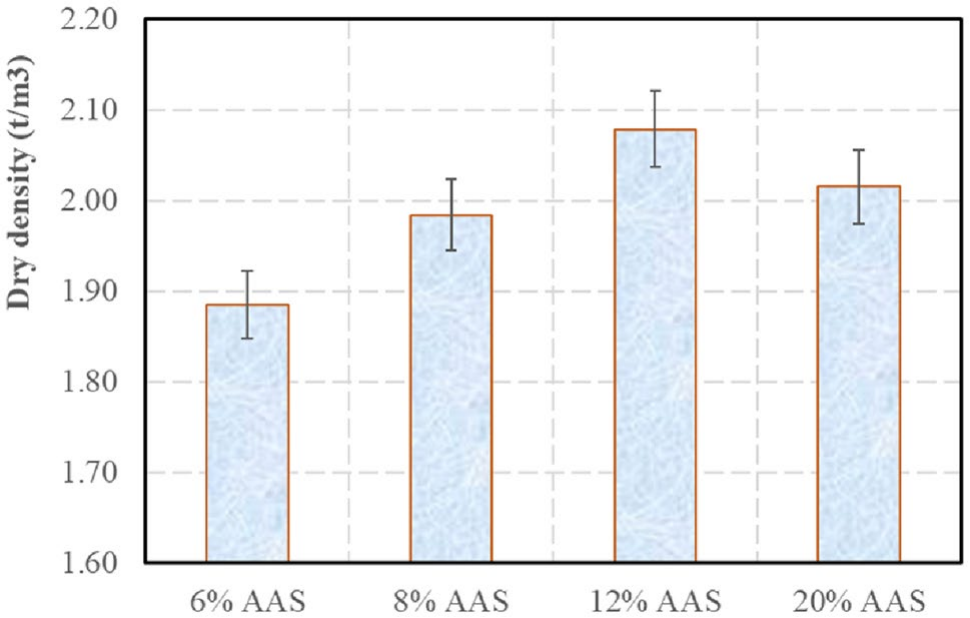
Dry densities of the blocks.

When compared to other previous studies on the CEB^26,27^, the compressive strength obtained in the present study was significantly higher. One of the reasons was that the dry density in the present study (about 1.98 t/m^3^ for 8% AAS, Fig. 8) was much higher than that of the previous studies with CEB (about 1.85 t/m^3^). This result shows the robustness of the mode of manufacturing used.

From Fig. 7, when the AAS amount increases, the compressive strength increases. However, when AAS amount increased from 6 to 12%, the compressive strengths increased significantly (about 400%), while when AAS amount increased from 12 to 20%, the enhancement of the compressive strengths was less significant (around 20%). It is first suggested that when AAS less than 8%, the developed geopolymer binder could not cover all particles. Moreover, the blocks of 12% AAS had highest dry densities (Fig. 8). The amount of AAS higher than this value increased the geopolymer in the blocks but may reduce the dry density of the blocks, because the liquid amount was higher than the optimum water content. The liquid amounts of the blocks of 6%, 8% and 12% AAS were similar, however, among these amounts, when AAS increased, the crystal amount increased also (due to the geopolymerisation). For 20% AAS blocks, although the liquid content was significantly higher than the optimum water content, the higher geopolymerisation in these blocks enhanced the dry density, which were similar to the highest value of 12% AAS blocks.

### Compressive strength in the saturated state

**Figure 9:**
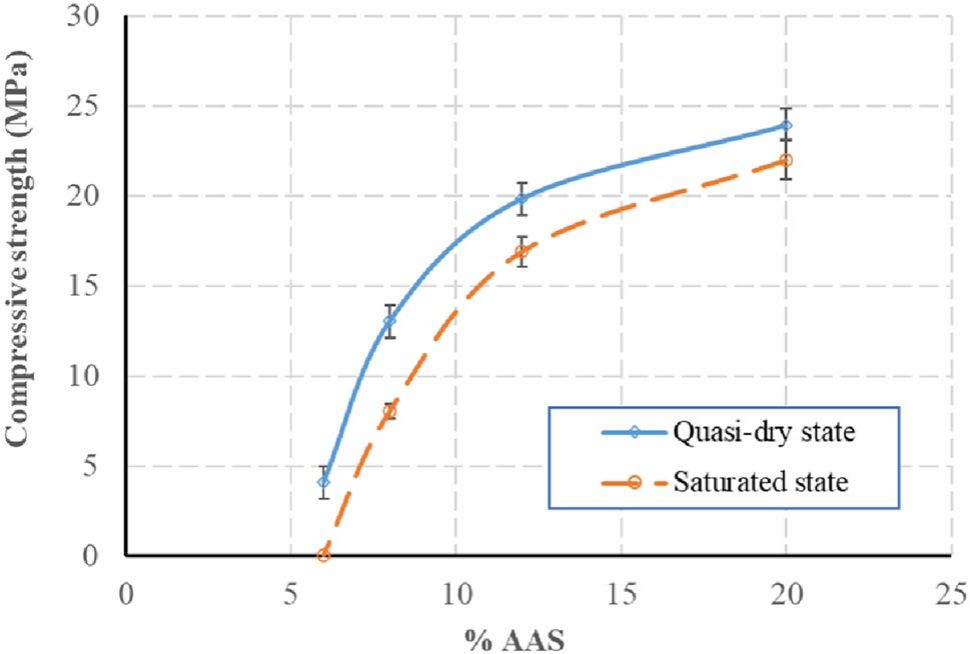
Compressive strength at the quasi-dry state and saturated state, at 28 days.

For the saturated compressive strength, the present study concentrated on the blocks cured under ambient conditions. The result of the saturated compressive strength is presented in Fig. 9. For the blocks of 6% AAS, the blocks were disintegrated after the immersion in the water. For the blocks stabilised from 8% AAS, the ratios of the saturated compressive strength on the quasi-dry compressive strength (*f*_*c,saturated*_*/f*_*c,dry*_) were all higher than 0.5. The difference in results of compressive strength in the quasi-dry state and in the saturated state is due to the difference of the suction in the samples in these states. The suction has been investigated and discussed in several previous studies^1,15^.

The comparison of the compressive strengths (in quasi-dry or saturated states) obtained from different studies is presented in Table 4. The studies presented in this table have the similar block dimensions. However, the slenderness ratio which is an important factor is different in some studies^17,18^. The effects of the slenderness ratio were discussed in detail in Aubert et al.^54^: for a same materials, the compressive strength can vary from 4.4 to 6.3 MPa for a slenderness ratio of 2 to 45 MPa for a slenderness ratio of 0.35. Therefore, the slenderness ratio of 2 is usually considered as reference because the influences of the friction between the block and the testing press is reduced^54^.

**Table 4:** Compressive strengths obtained from different studies.

References	Block dimensions (mm)	Stabilisation (in mass)	Manufacturing pressure	Manufacturing water content (%)	Slenderness ratio	Quasi-dry compressive strength (MPa)	Saturated compressive strength
Muntohar^17^	55 × 110 × 230	Lime and rice husk ash, until 15% lime	Compression 15 MPa by press	19	0.5	11–20	10–16 MPa
Oti et al.^18^	65 × 102 × 215	Ground granulated blastfurnace slag and until 3% lime	Compression 15 MPa by press	4.5–10	0.64	3.5–7	Not available
Villamizar et al.^19^	80 × 150 × 320	Coal-ash and Cassava peels	Compression by a machine (CEB)	29–39	1.9	1–3	Not available
Reddy and Latha^20^	75 × 108 × 230	4–10% cement	Compression by a machine (CEB), 0.3 MJ/m^3^	12.5–18	2	2.4–10	1–6 MPa
Aubert et al.^54^	48 × 136 × 407	Unstabilised	Manual compression (Adobe), dried progressively from 25 to 100 °C	16–18	0.35	45	Not available
	50 × 50 × 100				2	4.4–6.3	Not available
Present study	60 × 120 × 240	8% AAS	Manual compression (adobe)	10.5	2	13	8 MPa

It is observed from Table 4 that: although the blocks in the present study have been manufactured by a simple manual compression (and stabilised at 8% AAS), the compressive strengths obtained were the highest when compared to other studies. This result show the potential application of geopolymer stabilisation for adobe technique.

### Water absorption

**Figure 10:**
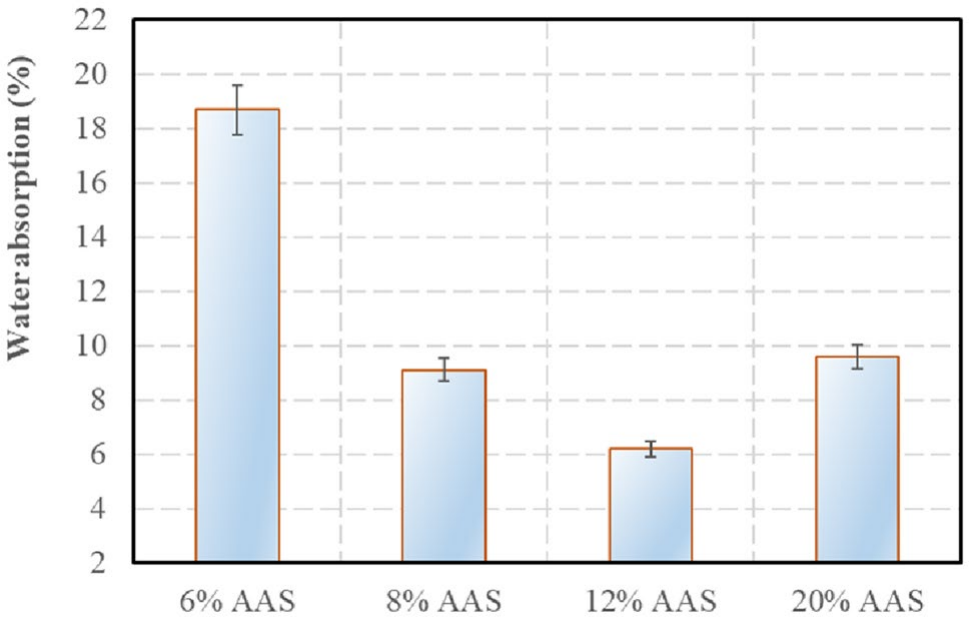
Water absorption of the blocks.

The result of the compressive strength at the saturated state is presented in Fig. 10. Following the standard^55^, the upper limit of the water absorption is 12% for the blocks having a mean compressive strength more than 5 MPa, and this limit is 14% for the blocks having a mean compressive strength less than 5 MPa. So, the blocks stabilised at 6% AAS could not satisfy this criterion about the water absorption, while other compositions satisfied this criterion. From Figs. 8 and 10, it is observed that there is a relationship between the water absorption and the dry density. Indeed, the water absorption is influenced by the porosity of the sample which is a function of the dry density. When the dry density increases, the water absorption decreases.

### Flexural tensile strength

**Figure 11:**
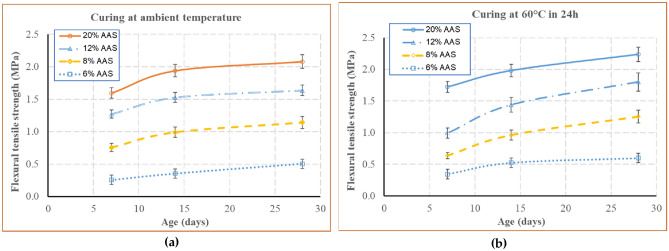
Flexural tensile strength as a function of time for the blocks cured at ambient temperature (**a**) and at 60 °C during 24 h (**b**).

The synthesis of the results on the flexural tensile strength of the blocks is presented in Fig. 11. This figure shows the evolution of the mean flexural tensile strength of the blocks in functions of time. It is observed that the strength developed rapidly until 14 days, then decelerated from this time. The chemical reactions to create geopolymer occur strongly in the first days after the manufacturing, then these reactions decelerate. This phenomenon leads to the rapid development of the geopolymer strength during the first days and then a slow-down from the 14th day^39,47^. Therefore, the geopolymer stabilised adobes have the similar trend. The blocks cured under 60 °C for 24 h had higher strength than the blocks cured under ambient temperature about 5%.

### Relationship between the compressive strength and the flexural tensile strength

**Figure 12:**
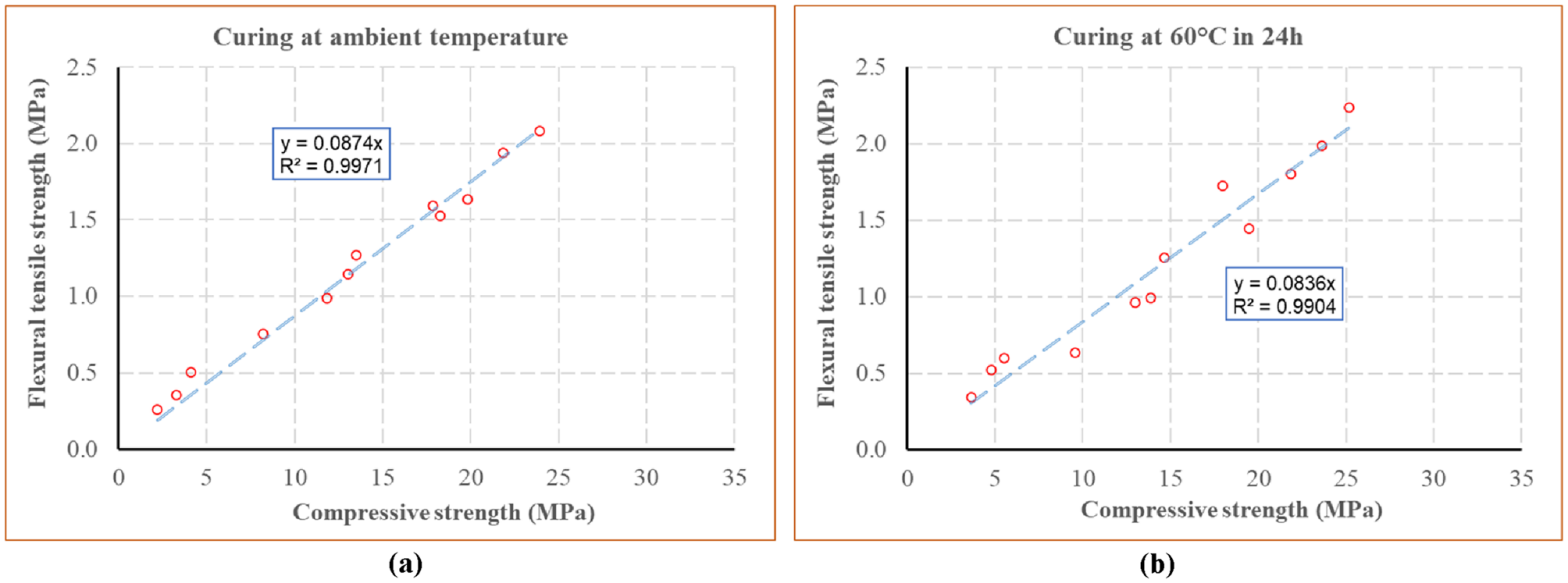
Relationship between the flexural tensile strength and the compressive strength for the blocks cured at ambient temperature (**a**); at 60 °C during 24 h (**b**).

The relationships between the mean flexural tensile strength (*f*_*tm*_) and the mean compressive strength (*f*_*cm*_) for the blocks cured at ambient temperature and at 60 °C during 24 h are presented in Fig. 12a,b, respectively. It is observed that *f*_*tm*_ was 8.7% and 8.4% of the *f*_*cm*_ respectively for these two cases. Therefore, a mean relationship can be written for these blocks:

$$ f_{tm} = \, 0.0{85}f_{cm} . $$

This relationship is similar to that presented some previous studies^56,57^ where *f*_*tm*_ was proposed to be 7–8% of *f*_*cm*_. However, for other studies^27,31^, higher values of *f*_*tm*_ were observed where *f*_*tm*_ was about 20% of *f*_*cm*_.

### SEM results

The micro-structure of the blocks stabilised at 20% AAS and at 8% AAS were analysed in detail respectively. The 20% AAS stabilised blocks had highest mechanical characteristics. The 8% AAS stabilised blocks had characteristics satisfying the exigencies of the existing standards (about the minimum compressive strength, the maximum water absorption, and the minimum ratio of saturated compressive strength on dry compressive strength), and the compromise between the technical and economic criteria.

**Figure 13:**
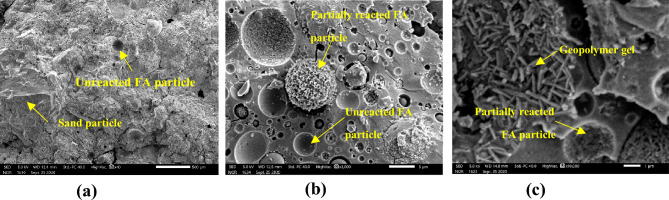
Microstructure of a block stabilised at 20% AAS. (**a**) Zoom at 500 µm; (**b**) zoom at 5 µm; (**c**) zoom at 1 µm.

**Figure 14:**
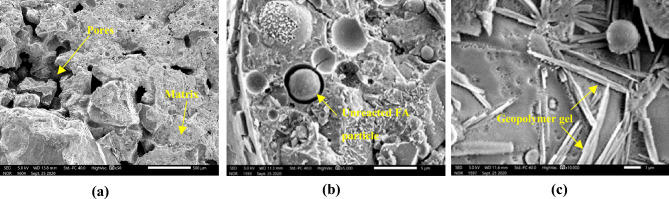
Microstructure of a block stabilised at 8% AAS. (**a**) Zoom at 500 µm; (**b**) zoom at 5 µm; (**c**) zoom at 1 µm.

SEM morphology of a 20% AAS stabilised block cured at ambient temperature for 28 days, is presented in Fig. 13. SEM morphology of a 8% AAS stabilised block cured at ambient temperature for 28 days, is presented in Fig. 14. From Fig. 14a, there were more micro-pores in the 8% AAS stabilised blocks than that of 20% AAS stabilised ones (Fig. 13a). The higher quantity of micro-pores can explain the lower mechanical characteristics of 8% AAS blocks when compared to 20% AAS blocks. It is suggested that with higher AAS mount, the AAS in 20% AAS blocks could better fill the micropores, then, by reactions between AAS and FA, the crystals could be created, which filled the micropores and did not evaporate during the curing. The creation of the geopolymer gels can be observed in Figs. 13b,c and 14b,c. It can be observed that numerous FA particles were only partially reacted with AAS to create geopolymer gels. It was noted for geopolymer-based materials (for example geopolymer concrete) that FA particles were usually not completely reacted, and could play the role as fillers^39^. So, with a higher FA amount, the 20% AAS blocks were also better filled. The presence of the geopolymer gels will be verified in the next section, by using the FTIR technique.

### FTIR results

**Figure 15:**
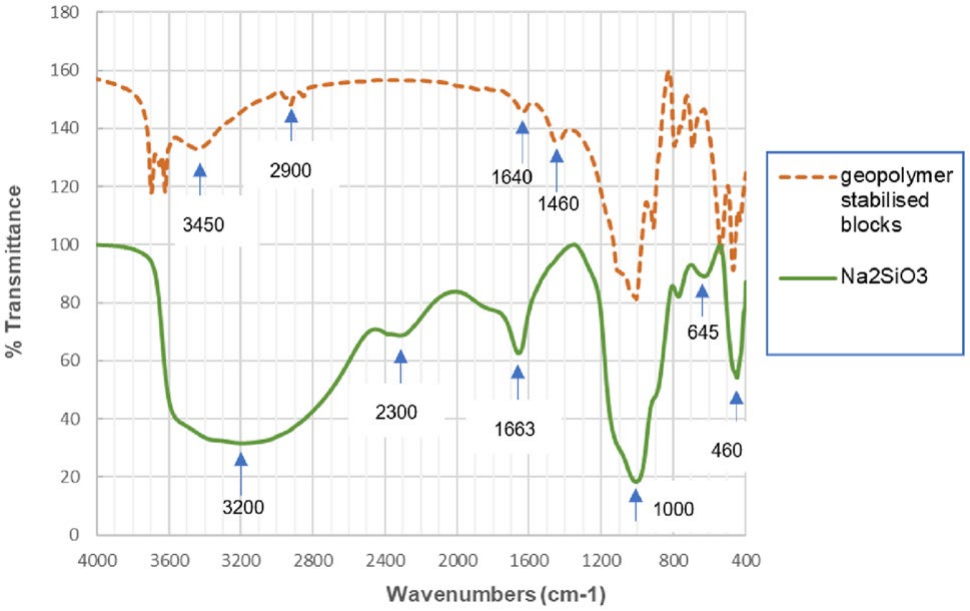
FTIR result of Na_2_SiO_3_ and a block stabilised at 8% AAS at 28 days.

The results obtained from FTIR technique on Na_2_SiO_3_ and on a 8% AAS stabilised block are presented in Fig. 15. The comparison between the spectrum of Na_2_SiO_3_ (before any reaction with NaOH and FA) and that of a block stabilised (after the reactions) enabled to verify whether the geopolymerisation occurred.

The FTIR spectrum of Na_2_SiO_3_ had two strong peaks: the first one at 1000 cm^−1^ of wavenumbers, and the second one was a large band from 2800 to 3600 cm^−1^. There were also other small peaks at the wavenumbers of 2300 cm^−1^, 1660 cm^−1^, 1120 cm^−1^, 900 cm^−1^, 780 cm^−1^, 650 cm^−1^ and 460 cm^−1^. These peaks are typical for the FTIR spectrum of Na_2_SiO_3_^58^.

When compared to the spectrum of Na_2_SiO_3_, the change of several peaks was observed for the FTIR spectrum of the 8% AAS block. The disparition or apparition of the peaks signifies the changes of the molecular bonds. In the spectrum of 8% AAS block, the peak at 2300 cm^−1^ (of Na_2_SiO_3_) disappeared, which indicated that there were the reactions between Na_2_SiO_3_, NaOH and FA. On the other hand, the new clear peaks appeared at 3450 cm^−1^ for 8% AAS block; these peaks corresponded to –OH and H–O–H bonds stretching vibration. The peak at 1663 cm^−1^ (corresponding to “not bonded water H-HO”) in Na_2_SiO_3_ moved to 1640 cm^−1^ (corresponding to “constitutional water”) for 8% AAS block. These peaks are the indicator of the hydration of geopolymer. The apparition of peaks at 1460 cm^−1^ in 8% AAS block confirms again the creation of geopolymer^59^.

For 8% AAS block, the small new peaks were also observed at the wavenumbers of 2900 cm^−1^. This wavenumber corresponded to the chemical bond of C–H^59^. This chemical bond could be created by the reactions between the unreacted activator with CO_2_ in the air^31^. The reaction of remaining activator with CO_2_ in the air was also observed in previous studies^59,60^ in which the peaks at 1430–1460 cm^−1^ appeared. The peak at 1460 cm^−1^ was also observed for the spectrum of 8% AAS block in the present study. This peak corresponded to the O–C–O stretching vibration of a carbonate phase, which confirms the reaction of the block with CO_2_. This reaction may be a supplementary interest of geopolymer-based materials because the reactions with CO_2_ can reduce the CO_2_ in the environment.

## Conclusion and outlook

The present paper investigated the geopolymer stabilisation for a sandy soil using the manual compression similar to the traditional adobe technique. The geopolymer was obtained by using the fly ash and the alkaline activator solution which was the mixture of Na_2_SiO_3_ liquid and NaOH solution. The AAS amounts were chosen at 6%, 8%, 12% and 20% in mass, respectively. The blocks had the dimensions of 60 mm × 120 mm × 240 mm.

The results showed that with the sandy soil used in the study, the compressive strength of the blocks tested were 13 MPa for 8% AAS which was higher than that obtained in the previous studies using clayey soils (10 MPa). This result suggests that the FA based geopolymer stabilisation may be more suitable for sandy soils than for clayey soils. The comparison with previous studies on soil blocks with similar dimensions showed that with a simple manual compression and 8% AAS stabilisation, the adobes obtained had highest values of compressive strengths (in quasi-dry or saturated states). This result shows the relevancy of the geopolymer stabilisation for adobe technique.

The tensile strengths of the blocks were also determined by using 3-point bending tests. The relationship between the flexural tensile strength and the compressive strength was identified. The SEM and FTIR techniques were applied to investigate the microstructure of the blocks. The geopolymerisation was confirmed by using the results obtained from these tests.

The results showed that 8% AAS blocks could satisfy different requirements of the current standards: the minimum saturated compressive strength, the maximum water absorption. With the same stabiliser amount, geopolymer stabilised material has lower carbon footprint than that of cement stabilised, which shows the promising application of geopolymer stabilised adobes.

Further studies on other properties of the blocks obtained, such as the Young’s modulus, the shrinkage and creep phenomena will be interesting. Then, studies on the behaviour of walls (made from geopolymer stabilised adobes and mortar) will be necessary. The thermal properties and the hygro-thermal characteristics of geopolymer stabilised adobes are also the interesting topics for further studies.

## Data Availability

The data presented in this study are available on request from the corresponding author.
